# Importance of *N*-Glycosylation on CD147 for Its Biological Functions

**DOI:** 10.3390/ijms15046356

**Published:** 2014-04-15

**Authors:** Yang Bai, Wan Huang, Li-Tian Ma, Jian-Li Jiang, Zhi-Nan Chen

**Affiliations:** Cell Engineering Research Centre and Department of Cell Biology, State Key Discipline of Cell Biology, Fourth Military Medical University, Xi’an 710032, Shaanxi, China; E-Mails: sydbaiyang@163.com (Y.B.); huangwan@fmmu.edu.cn (W.H.); malitian1234@163.com (L.-T.M.)

**Keywords:** CD147, *N*-glycosylation, glycosyltransferases, matrix metalloproteinase, cancer invasion and metastasis

## Abstract

Glycosylation of glycoproteins is one of many molecular changes that accompany malignant transformation. Post-translational modifications of proteins are closely associated with the adhesion, invasion, and metastasis of tumor cells. CD147, a tumor-associated antigen that is highly expressed on the cell surface of various tumors, is a potential target for cancer diagnosis and therapy. A significant biochemical property of CD147 is its high level of glycosylation. Studies on the structure and function of CD147 glycosylation provide valuable clues to the development of targeted therapies for cancer. Here, we review current understanding of the glycosylation characteristics of CD147 and the glycosyltransferases involved in the biosynthesis of CD147 *N*-glycans. Finally, we discuss proteins regulating CD147 glycosylation and the biological functions of CD147 glycosylation.

## 1. Introduction

CD147, a type-I transmembrane glycoprotein of the immunoglobulin superfamily (IgSF), was originally purified from the plasma membrane of the human LX-1 lung carcinoma cell line in 1982 [1]. It is found expressed in various cells, including platelets, fibroblasts, T-lymphocytes, and, especially, in tumor cells [2–4]. It has many designations across species including human extracellular matrix metalloproteinase inducer (EMMPRIN) [5], hBasigin [6], M6 [7] and HAb18G [8]; rat OX-47 [9] and CE9 [10]; mouse gp42 [11] and basigin-1 [12]; and chicken HT7 [13], neurothelin [14], and 5A11 [15]. Numerous studies have documented the significance of CD147 in various physiological processes, such as spermatogenesis and fertilization, neural network, and retinal development [12,16–18], and in the progression of several diseases including atherosclerosis, rheumatoid arthritis, and infections by malaria parasites and virus [19–22]. The best characterized function of CD147 is its role in tumor metastasis, angiogenesis, and chemoresistance [23–25]. As an adhesion molecule, CD147 mediates molecular events by interacting with a wide range of tumor and inflammation-associated molecules including integrins [24], monocarboxylate transporters (MCTs) [25], cyclophilins [26], caveolin-1 [18], and E-selectin [27].

Alteration in glycans of glycoproteins and glycolipids is a significant characteristic of tumor malignant transformation, and is closely associated with the adhesion, invasion and metastasis of tumor cells [28]. CD147 is post-translationally modified through *N*-glycosylation. Investigations into CD147 glycosylation have clarified its role in numerous physiological and pathological events. This review focuses on recent progress on the structural and biological characteristics of CD147 glycosylation and recapitulates glycosyltransferases involved in the biosynthesis of CD147 asparagine-linked oligosaccharides (*N*-glycans) to seek future therapeutic strategies for CD147-associated diseases.

## 2. Structure of CD147

CD147 consists of a 21 amino acid (aa) signal peptide, a 185 aa extracellular domain (ECD), a 24 aa transmembrane domain, and a 39 aa cytoplasmic domain [5]. Four cysteines (41, 87, 126, and 185) located in the extracellular region form two typical IgSF domains [6], which share deep homology to the IgVκ domain and the β-chain of MHCII antigens [29]. The *N*-terminal domain is responsible for counter receptor activity and protein oligomerization [30,31]. The *C*-terminal domain is responsible for association with caveolin-1 [32], integrins (α3β1 and α6β1) [24,33], and annexin II [34]. The transmembrane domain possesses a series of conserved hydrophobic amino acids except a rarely occurring charged glutamic acid (218) in transmembrane proteins [5]. The transmembrane domain exhibits affinity toward other proteins, such as cyp60 [35], CD43 [36], and syndecan [37], thus, eliminating the high-energy charge. Moreover, it possesses a typical leucine zipper motif containing three leucines (206, 213 and 220) and a phenylalanine (227) appearing at every seventh residue, which facilitates membrane-protein associations and diverse cellular signal pathways [9,13]. The highly conserved intracellular domain of CD147 plays a pivotal role in association with MCTs (MCT1, MCT3 and MCT4) [38], although it has not been well explored.

Alternative splicing and alternative promoters result in four isoforms of CD147. Among them, basigin-1 is a retina specific CD147 containing an additional unglycosylated domain [39,40]; basigin-3 or basigin-4, less expressed in normal and tumor human tissues, contains a single extracellular domain (IgI), and basigin-3 serves as an endogenous inhibitor of basigin-2 via hetero-oligomerization. Both basigin-3 and basigin-4 have HG (highly glycosylated) and LG (lowly glycosylated) forms as observed in basigin-2 [41]. However, the knowledge of glycosylation of the above-mentioned scarce isoforms is limited. Given that the ubiquitously expressed basigin-2 mediating matrix metalloproteinases (MMPs) production is profoundly explored, we will concentrate on the glycosylation of basigin-2 in the following discussion.

**Figure 1.**
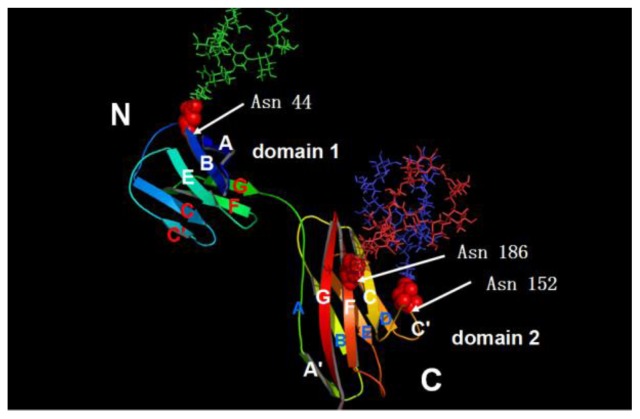
Molecular model of the extracellular region of CD147 and its three glycosylation sites. The *N*-terminal domain is a typical C2 set immunoglobulin domain consisting of a β-barrel formed by the sheets EBA and GFCC′ and a conserved disulfide bond between strands B and F. The *C*-terminal domain is a typical I set immunoglobulin domain formed by the β-sheets DEBA and A′GFCC′ and a disulfide bond between Cys126 and Cys185 connecting strands B and F together. One *N*-linked glycosylation site, Asn44, lies at the end of strand B, *i.e.*, the outermost position of the EBA sheet. The other two sites, Asn152 and Asn186, locate at the middle of C′D loop and strand F, respectively, with their lateral chains protruding oppositely from A′GFCC′ and DEBA sheets [42]. The figure is generated using the GlyProt software program [46,47] and we select oligomannoses on behalf of the potential diverse glycan structures to create the 3D protein structure of CD147.

The crystal structure of the ECD of CD147 (Figure 1) was revealed by X-ray analysis [42]. CD147 crystallizes in space group with four monomers in the asymmetric unit. Each monomer consists of a typical *N*-terminal IgC2 set immunoglobulin domain 1 (22–101) and a typical *C*-terminal IgI set immunoglobulin domain 2 (107–205), which are connected by a 5 aa flexible linker responsible for diverse inter-domain angles within the four monomers. This unique C2-I domain arrangement distinguishes CD147 from all other IgSF proteins with known structures. With edge-by-edge packing and association of β-sheets, monomer interaction leads to two types of dimerization: C2–C2 dimerization (BC, AC and DD′ dimers) symbolizing a *trans*-cellular homophilic interaction between two CD147 molecules on neighboring cell membranes and C2-I dimerization (AD dimer) representing a heterophilic interaction between CD147 and other IgSF proteins. These dimers further adhere to each other by sharing some conserved β-strands at either edge of the β-barrels [42]. A further structure analysis of domain 1 illustrated that it formed a dimer through the exchange of its β-strand (strand G) [43]. Oligomerization contributes to CD147’s functions, including counter-receptor binding, association with other proteins, and MMPs induction [44,45].

## 3. The Glycosylation Characteristic of CD147

The overwhelming majority of studies showed that CD147 is a *N*-linked glycosylated protein except one study by Fadool *et al.*, which demonstrated that chicken 5A11/HT7 antigen of neural retina and epithelial tissues contains both *N*-linked and *O*-linked oligosaccharides [13]. Members of CD147 family, EMMPRIN, basigin, 5A11/HT7 for instance, from different species, tissues or cells appear as diverse glycosylated forms, with large variation in molecular weight [32,48–50]. In this review we focus on the *N*-glycosylation of CD147. The unglycosylated CD147 has a molecular weight of 27 kDa, whereas the glycosylated form has a molecular weight between 43 and 66 kDa [7,15,48–50]. Treatment with different endoglycosidases indicates that *N*-glycans contribute to almost half the size of the mature molecule [7,51].

Combining with site-specific mutagenesis study, the sequence alignment demonstrated that there were three conserved Asn glycosylation sites across species in the ECD of CD147 [5,32,42]. Mutation of three *N*-glycosylation sites (N44Q, N152Q, and N186Q) caused an approximately equal decrease in the molecular weight of HG-CD147 and LG-CD147, suggesting that they make comparable contributions to CD147 glycosylation [32]. The study unraveling the crystal structure of CD147 provided the proof of the spatial position of three glycosylation sites: Asn44 at the end of strand B, Asn152 and Asn186 at the middle of C′D loop and strand F (Figure 1), respectively [42].

### 3.1. HG-CD147 and LG-CD147

**Figure 2.**
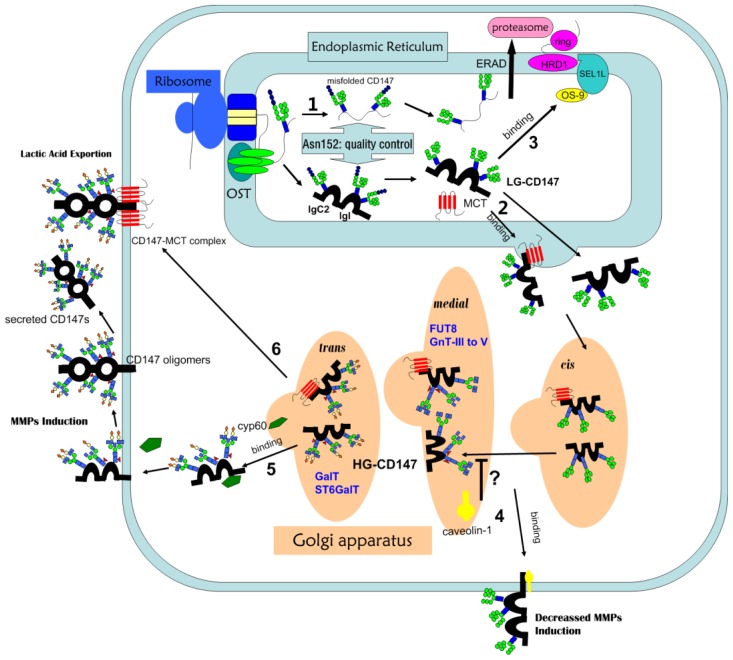
Intracellular biosynthesis and trafficking of glycosylated CD147. Immature high-mannose form of CD147 is modified in the ER, during which **1** glycans on the Asn152 are essential for quality control. Misfolded proteins without Asn152 glycosylation are degraded through ERAD pathway [53]; **2** A part of LG-CD147 then enter the Golgi while **3** the majority of newly produced LG-CD147 are degraded by the proteasome via the OS-9/SEL1L/Hrd1 pathway [54]. In the Golgi complex, LG-CD147 is further modified by many glycosyltransferases including GnT-III, GnT-IV, GnT-V and FuT-8 to form more complicated branching carbohydrate chains [53,55]. Subsequently, terminal modifications such as sialic acids are added to CD147 [56]; **4** Caveolin-1 binds to LG-CD147 in the Golgi, inhibits its maturation and escorts it into the cell membrane. LG-CD147 on the membrane fails to self-associate and induce MMPs [32]. However, it is also reported that caveolin-1 facilitates CD147 maturation [57]; **5** Then, HG-CD147 translocates to plasma membrane during which cyp60 in the Golgi is one of chaperones facilitating the translocation of CD147 [35]. Mature CD147 on the cell membrane form oligomers and a small fraction of transmembrane CD147 are shed and released into the extracellular matrix to act on neighbouring cells. Both forms of mature CD147 are capable of inducing MMPs; **6** MCT is one of ancillary proteins that accompany CD147 during its maturation in the ER and they form CD147-MCT complex on the membrane bearing the double roles of MMPs induction and lactic acid exportion [58,59].

A distinct feature of CD147 from various cells and tissues is that based on the degree of glycosylation, two bands were shown on the result of Western blotting, suggesting that CD147 exists in two forms: HG-CD147 (~40–60 kDa) and LG-CD147 (core-glycosylated CD147, ~32 kDa). HG-CD147 contains complex-type carbohydrate that is sensitive to Peptide N Glycosidase F (PNGase F), whereas LG-CD147 contains high-mannose carbohydrate that is sensitive to Endoglycosidase H (endo H) [32]. In terms of the general process of protein glycosylation [52] and the characteristic of CD147 glycosylation [32,53], we may conclude that nascent peptide of CD147 receives preliminary glycosylation in the ER (Figure 2), forming an immature high mannose form (LG-CD147), and in the Golgi complex CD147 is further modified to form more complicated branching carbohydrate chains and specific terminal structures by a series of glycosyltransferases. Subsequently, the fully glycosylated mature CD147 (HG-CD147) is translocated to plasma membrane. In this context, LG-CD147 is the precursor of HG-CD147 in the ER, which requires an additional modification in the Golgi prior to express on the cell surface [32]. Different cell types have different HG/LG ratio and it is reported that both HG-CD147 and LG-CD147 could be detected on the plasma membrane [32], but there are also studies revealing that only fully glycosylated CD147 could be found on plasma membrane in hepatoma tumor cells [53] and COS-7 cells [30]. As a transmembrane protein, HG-CD147 on the plasma membrane is considered to be the biological functional form. Comparatively, whether LG-CD147 stably existing within the hepatoma tumor cells participates in other cellular physiological functions remains to be further investigated.

### 3.2. The Structure of the Oligosaccharides of CD147

CD147 is a transmembrane glycoprotein expressed on various tumor cells. Disclosing the structure of the oligosaccharides of CD147 from tumor tissues will provide valuable clues for the development of novel therapeutic modalities against tumor. However, due to the difficulties in purifying enough native transmembrane proteins from tumor tissues, determining the *N*-glycan profiles of CD147 by mass spectrometry analysis is a challenge. In a recent study, native CD147 was purified from lung carcinoma tissue specimen from a patient by immunoaffinity chromatography using mAbHAb18, and the structures of *N*-glycans of CD147 have been characterized by means of Nanospray Ionization-Linear Ion Trap (NSI-MS) [53]. The results showed that purified CD147 exhibited both high-mannose type and bi-antennary complex-type oligosaccharides, which was in accordance with the glycosidases treatment results of Yu *et al.* [51]. Moreover, the presence of β1,6-branched oligosaccharides on CD147 was confirmed by lectin blotting carried out with Phaseolus vulgaris Leukoagglutinin (L-PHA) [32,53]. Fan *et al*. found that Phaseolus vulgaris Erythroagglutinin (E-PHA) also bound to CD147 immunoprecipitated from mouse hepatocarcinoma cells, indicative of bisecting structures in *N*-glycans of CD147 [55]. In addition, these glycans can be fucosylated and sialylated. It is noteworthy that native CD147 from human lung cancer tissue contained a high percentage of core fucosylated structures (28.8%) [53]. Miyauchi *et al.* discovered that Lotus tetragonolobus agglutinin (LTA) bound to CD147 from embryonal carcinoma cells and Kato *et al*. found that CD147 served as a ligand for E-selectin which recognizes sialylated glycans, such as Lewis X (sLex), both implying the sialyl Lewis X structure, namely, Galβ1, 4Fucα1,3GlcNAc, in *N*-glycans of CD147 [27,29]. In addition, a further study by Yang *et al.*, who identified sialoglycoproteins in the cell surface of prostate cancer cell ML-2 by mass spectrometry analysis, also revealed that CD147 was one of the metastasis-related sialylated proteins [56]. Thus far, the existence of β1,2-branching structures in CD147 glycosylation has not been reported. In-depth mass spectrometry analysis, such as to characterize glycans on all the three *N*-glycosylation sites on CD147 and to disclose the differences of *N*-glycosylation between the CD147 from normal tissues *versus* tumor tissues will improve our understanding of the biological role of aberrant *N*-glycans on CD147 during cancer progression.

## 4. Glycosyltransferases Involved in the Modulation of CD147 *N*-Glycans

**Figure 3.**
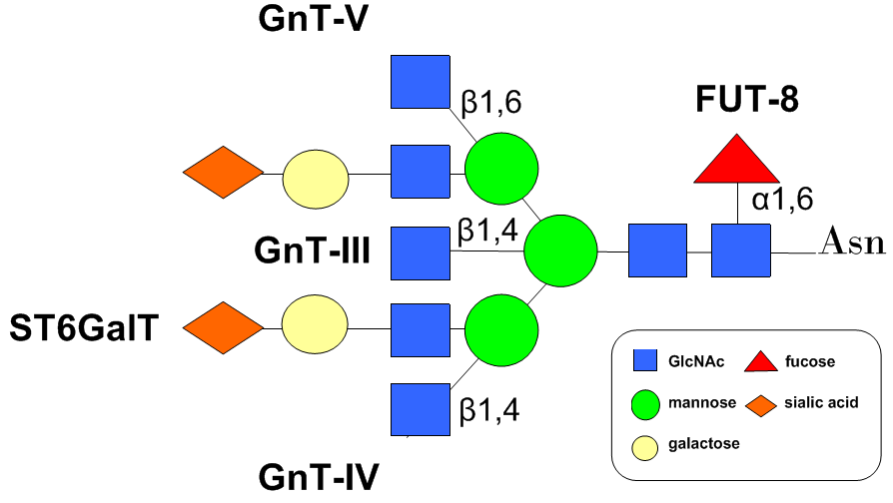
Potential glycan structures of CD147 and corresponding enzymes. In the medial-Golgi compartment, GnT-IV catalyzes β1–4 branch on complex *N*-Glycan structures, while GnT-III and GnT-V catalyze the formation of bisecting structure and β1–6 branch, respectively. The core fucose structure is catalyzed by FUT8. Then CD147 enters the *trans*-Golgi apparatus and receives sialic acid modification by sialyl transferase [53,55,56].

Branched *N*-glycans are biosynthesized by glycosyltransferases, such as GnTs (*N*-acetylglucosaminyltransferases), Futs (fucosyltransferases), GalTs (galactosyltransferases) and STs (sialytransferases) in the ER and the Golgi apparatus. Based on the *N*-glycan profiles of CD147 described above, many glycosyltransferases have been considered to play important roles in the biological functions of CD147 (Figure 3).

The absence or redundancy of glycosyltransferases may produce abnormal carbohydrate chains. The modulation of the *N*-glycans of cancer-associated proteins by these enzymes alters cell behaviors, such as cell signaling and cell adhesion, which are implicated in tumor invasion and metastasis [60]. In terms of CD147’s functions during tumor progression, the colorectal carcinoma progression is owing to the up-regulation of CD147 without the alteration of its glycosylation [61]; however, in other conditions, researchers observed the anomalous glycosylation or the combination of the two changes [57,62–64]. Considering this, both the quantity and the quality of CD147 should be taken into consideration, and the aberrant glycosylation of CD147 by corresponding enzymes may deserve more attention during tumor metastasis.

### 4.1. GnTs

As key glycosyltransferases regulating the formation of periphery multi-antennary structures, members of GnT family facilitate the formation of the *N*-linked oligosaccharides from the high mannose type to the complex type through the hybrids type by adding *N*-acetylglucosamine (GlcNAc) antennaes in the medial-Golgi apparatus [65]. The roles of GnT-III, GnT-IV, and GnT-V in CD147 glycosylation will be discussed in the following part, but it has not been reported yet whether GnT-I and GnT-II both catalyzing β1,2GlcNAc branch formation, GnT-VI catalyzing β1,4GlcNAc branch formation, and GnT-IX catalyzing β1,6GlcNAc branch formation [52] participate in CD147 glycosylation.

GnT-V, located in the medial/trans Golgi, catalyzes the formation of β1,6GlcNAc branch on the trimannosyl terminus of *N*-glycans, the product of which can be further extended with poly-*N*-acetylgalactosamine (GalNAc) chains and then terminally modified with sialylated structures [66]. Overexpression of GnT-V in tumor cells leads to aberrant β1,6-branching, which contributes to tumor progression [67]. To be specific, increased β1,6-branching of *N*-linked glycans is highly associated with various biological functions of some molecules, thereby affecting cancer metastasis. E-cadherin, integrins, matriptase and TIMP-1 (tissue inhibitor of matrixmetalloproteinase-1) are representative molecules glycosylated by GnT-V [68–71]. In a recent study, it was evidenced that GnT-V is crucial for the function of CD147 in SMMC-7721 cells. Functional studies in GnT-V over-expressing cells showed a significant increasing in MMP-2 activity. Moreover, the results also indicated that CD147 is a target protein of GnT-V through which GnT-V promotes tumor metastasis [53].

GnT-III catalyzes the addition of bisecting GlcNAc structures to *N*-glycans via β1,4-linkage, the product of which suppresses the action of GnT-V, thus preventing the metastatic capability [72]. A previous study suggested the existence of bisecting structures in *N*-glycans of CD147 in mouse hepatoma cells, indicating that GnT-III may be involved in the glycosylation of CD147 [55], so its role in the biological functions of the protein merits further exploration.

GnT-IV transfers the β1,4GlcNAc branch on the core structure of *N*-glycans, the product of which is a substrate for GnT-III and GnT-V [73,74]. Both hepatoma and choriocarcinoma tissue represented an up-regulated GnT-IV activity, and human chorionic gonadotropin (hCG) from choriocarcinoma exhibited aberrant β1,4GlcNAc branch, suggestive of the role of GnT-IV during tumorigenesis [75–77]. Fan *et al.* found that up-regulated expression of GnT-IVa (an isoenzyme of GnT-IV) in Hepa1–6 cells increased the antennary branches and reduced bisecting branches of the *N*-glycans of many proteins, thus enhancing tumor migration. Overexpression of GnT-IVa also increased the HG/LG ratio of CD147 and changed the antennary oligosaccharide structures on CD147 in mouse hepatoma cell lines, suggesting that CD147 may be a target protein through which GnT-IVa modulates tumor metastasis [55].

### 4.2. FUT8

Core fucosylation (α1,6-fucosylation) is catalyzed by fucosyltransferase 8 (FUT8) which adds a fucose residue to the reducing terminal GlcNAc of the core structure on *N*-glycans via α-1,6 linkage. Core fucosylated proteins play an essential role in tumorigenesis, tumor invasion and angiogenesis [78,79]. The α1,6-fucosylation is essential for integrin α3β1 and E-cadherin mediated cell migration and enhances epidermal growth factor receptor (EGFR) mediated cell invasion by promoting its dimerization and phosphorylation [80–82]. The aberrant α1,6-fucosylation of molecules, such as CK8, annexin I, and annexin II, is involved in the metastasis of hepatocellular carcinoma [83]. The results from NSI-MS analysis of the *N*-glycans of CD147 revealed a high percentage of core fucose structure in human non-small cell lung cancer (NSCLC) tissue [53], suggesting a plausible role of fucosylated CD147 in tumor invasion, which could be a potential indicator for the prognosis of NSCLC.

### 4.3. Sialyltransferase

Sialic acid is a kind of acidic monosaccharide typically found at the terminus of *N*-glycans, catalyzed by sialyltransferase in the *trans*-Golgi apparatus. Sialyltransferase catalyzes structures of numerous antigens, such as Tn (sTn), polysialic acid (PSA), and sLex, which have been adopted as an effective indicator in the clinical diagnosis of tumor [84–86]. As the ligand of selectin, sialyl acid modified antigens mediated the adhesion between tumor cell and other cell types, such as platelet, leukocyte, and vascular endothelial cell [87]. As mentioned above, *N*-glycans of CD147 contain sialyl Lewis X structure [27,29,56]. Functional importance of CD147 sialylation and fucosylation in cancer progression should be further explored.

## 5. Proteins Regulating the Glycosylation of CD147

The unique structure characteristic of CD147 facilitates its interactions with various proteins such as cyclophilins, MCTs, presenilins, and caveolin-1. Some proteins have been well accepted as regulators in the process of CD147 maturation and translocation to cell surface (Figure 2).

As a scaffolding protein, caveolin containing cholesterol and glycosphingolipid components within the plasma membranes mediates processes such as caveolae biogenesis, transmembrane transport, signal transduction, and tumorgenesis [88]. Intriguingly, its role in regulating the conversion of LG-CD147 to HG-CD147 is inconsistent. By binding to the IgI domain of CD147, caveolin-1 associates with LG-CD147 during glycosylation process in the Golgi apparatus and escorts it to the plasma membrane, thus inhibiting the conversion of LG-CD147 to HG-CD147 and CD147 oligomerization at the cell membrane [32,89]. Furthermore, caveolin-1 associates with GnT-III and regulates its localization within the Golgi complex, which enhances GnT-III’s activity and, hence, prevents the action of GnT-V [90]. However, there is no direct evidence indicative of functional interaction between caveolin-1 and CD147 in normal and bleomycin-induced rat fibrotic alveolar cells [91]. In addition, Jia *et al.* has demonstrated that caveolin-1 enhances the HG/LG ratio and invasive ability of mouse hepatoma cells [57], suggesting the dual character of caveolin-1 in tumor migration. Apart from enhancing β1,6-branching in complex and hybrid *N*-glycans [57], caveolin-1 also up-regulates α-2,6-sialyltransferase I (ST6Gal-I) expression and then promotes the α2,6-sialylation of integrin, thus increasing tumor cell adhesion to extracellular matrix (ECM) [88,92].

Apart from caveolin-1, MCTs are also regarded as regulators of the glycosylation and trafficking of CD147. Tumor cells exhibit a high rate of glycolysis under both oxygen deficit and enriched circumstances to guarantee continuous energy supply and immoderate tumor growth, respectively. The metabolic byproducts of glycolysis, for example, lactic acid, accumulate in the cytoplasm and trigger apoptosis. MCTs mediate proton-coupled transportation of monocarboxylic acids and glycolytic byproducts out of the cells. The secreted lactates contribute to an acid microenvironment, which promotes invasion, metastasis and drug resistance of tumor cells [93]. As a chaperone, CD147 tightly binds to MCTs (MCT1, MCT3, and MCT4) in the ER during their trafficking to the cell surface, and forms a functional complex with them on the membrane by which MCTs mediate the transportation of monocarboxylic acids [58,94–96]. On the other hand, CD147 maturation is also dependent on its association with MCTs. Knocking down MCT4 in breast cancer cells and MCT1 in intestinal epithelial cells both led to the reduction in the expression of fully glycosylated CD147 and the accumulation of core-glycosylated CD147 in the ER, implicating that MCTs (MCT1, MCT4) regulate the maturation and trafficking of CD147 [58,59]. Above all, MCTs and CD147 cooperate with each other to enhance tumor progression through creating acid microenvironment and the degradation of ECM.

In addition, it is reported that cyp60, a member of the cyclophilin family serving as receptors for the immunosuppressive drug cyclosporin A (CsA) and regulating protein trafficking [26], is a chaperone during the transportation of CD147 from the lumen of Golgi to the plasma membrane by binding to the Pro211 at the interface between the transmembrane and extracellular domains of CD147 [35,97].

Amyloid β-peptide (Aβ) sedimentation is significantly implicated in the progression of Alzheimer’s disease (AD). It is produced from amyloid precursor protein (APP) after sequential proteolytic processes by β- and γ-secretase. γ-Secretase is a multimeric aspartyl protease consisting of at least four subunits, among which presenilin-1 or -2 (PS1 or PS2) provides the catalytic aspartyl residue [98]. Recent studies showed that as a γ-secretase associated protein, CD147 was up-regulated in several brain tissues of AD patients. Moreover, intracellular trafficking of CD147 was affected by PS2 [99,100]. The results of immunofluorescence staining suggested that in PS2-deficient cells, CD147 located around the nucleus instead of expressing on the cell surface, which was involved in the mechanisms of AD [100]. On one hand, the inhibition of CD147 maturation may reduce the production of MMPs and subsequent clearance of Aβ by proteolysis; on the other hand, since CD147 is a regulating subunit of γ-secretase, immature CD147 may attenuate γ-secretase activity and lead to Aβ sedimentation [99,100]. Detailed mechanisms underlying CD147’s association with γ-secretase in AD remain to be investigated.

## 6. Biological Role of CD147 Glycosylation

### 6.1. The Implication of HG/LG Ratio in Physiological and Pathological Processes

As an inducer of MMPs, CD147 participates in numerous physiological processes, and the glycosylation level of CD147 is regulated by the rhythm of hormones secretion. The HG/LG ratio significantly increases in chorio-decidua and amnion during term labor compared with nonlabor stage, with the total amount of CD147 remaining unchanged. CD147 together with subsequent MMPs production facilitates the placenta and fetal membranes to separate from the maternal uterus [101]. During the menstrual cycle, the expression and the glycosylation of CD147 in human endometrium exhibit a cyclical fluctuation and are enhanced by progesterone to degrade endometrial ECM in the secretion phase, which is an essential mechanism of menstrual endometrium remodeling [102].

Researchers have been concerned about its role in non-tumor diseases. The glycosylation of CD147 mediates IL-13 induced MMPs expression in epithelial airway cells through interaction with caveolin-1, triggering the development of asthma [103]. Different glycosylated forms of CD147 produce different types of MMPs, thus, determining the stability degree of atherosclerotic plaque, and HG-CD147 is associated with unstable plaque phenotype [104]. HG-CD147, together with MMP-1 expression, is also up-regulated in chronic periodontitis tissue [105].

Apart from non-tumor diseases, the HG/LG ratio also carries significant implications in neoplastic disease. Jia *et al.* found hepatoma carcinoma cell lines with higher lymphatic metastasis ability exhibited a higher HG/LG ratio than those with low or no lymphatic metastasis ability [106]. Moreover, Beesley and co-authors also found that HG-CD147 was closely related to acute lymphoblastic leukaemia and its relapse [62]. Aberrant glycosylation of CD147 is also involved in the multidrug resistance in human leukemia [107].

### 6.2. CD147 Glycosylation and MMPs Induction Activity

The role of *N*-glycosylation in CD147-dependent MMP production is controversial. Both purified glycosylated recombinant CD147 from CHO cells and purified native CD147 from tumor cells directly promoted MMPs production [31,108]. In addition, Sun *et al.* found that purified deglycosylated CD147 by tunicamycin treatment from HT1080 cells failed to produce MMP-1 and MMP-2 [31]. However, in contrast to Sun’s result, the unglycosylated recombinant CD147 obtained by Belton could bind to the CD147 on the surface of uterine fibroblasts, and then induce MMPs expression. This homo-interaction of CD147 was not dependent upon the glycosylation of CD147 ligand [109]. In a recent study, we compared the efficacy of glycosylated and unglycosylated CD147, and found that both produced MMPs, but eukaryotic native CD147 stimulated MMPs production more efficiently than prokaryotic recombinant CD147, convincing that carbohydrates do contribute to CD147’s activity [53].

The synthesis technique of peptide thioester carrying *N*-linked core pentasaccharide by Toole BP and co-authors provided an effective way to elucidate the role of CD147 glycosylation [110,111] and they demonstrated that IgC2 synthesized by the thioester method substituted with a chitobiose unit, IgC2-(GlcNAc)_2_, instead of IgC2 alone or the chitobiose unit alone, mimicked CD147’s MMP-2 induction capability in human fibroblast cells, with the underlying assumption that the hydrogen bonds between amino acids and the chitobiose unit may help preserve an active molecular conformation [112]. Toole also suggested another possible mechanism through which the glycosylation of CD147 engaged in MMPs production, that is, carbohydrate lateral chains of CD147 may be involved in its binding to the fibroblast receptor and subsequent signal transmission into the cell [113]. A recent study performed by Papadimitropoulou *et al.* comparing the MMP-2 induction ability of ECD, domain 1 and domain 2 of CD147 in both glycosylated and unglycosylated forms demonstrated that only glycosylated forms were able to stimulate MMP-2 production, further verifying *N*-glycosylation is a prerequisite for the activity of CD147 [114].

CD147, like other Ig-containing molecules, interacts homotypically. The role of glycosylation in the oligomerization of CD147 remains unsettled. Previous studies indicated that HG-CD147 instead of LG-CD147 became self-associated, which was demonstrated by anti-CD147 mAb immunoprecipitation, caveolin-1 treatment and covalent cross-linking agent treatment [32,89]. However, Yoshida *et al.* believed that *N*-glycosylation was not involved in homophilic cis-interaction of CD147 [30]. The crystal structure resolved by our lab revealed that the recombinant CD147 in crystal formed oligomers and the three glycosylation sites were distant from the dimer interface [42], suggesting a rare possibility that glycosylation participates in the oligomerization process. We further proved that Lys63 and Ser193 instead of the glycosylation sites were essential to CD147 dimerization [45]. Furthermore, the recombinant prokaryotic CD147 in solution were also oligomers [109]. However, Schlegel *et al.* demonstrated that extracellular domains of CD147 were monomeric in solution [115]. The results in our previous study proved that although prokaryotic CD147 could form oligomers in a glycan-independent manner at a low level, glycosylation could enhance the oligomerization of eukaryotic CD147 and all the native eukaryotic CD147 in solution formed oligomers [53]. The mechanism how glycosylation enhances the oligomerization of CD147 is unknown, and we reason that glycans stabilize the advanced protein conformation of CD147, which is an active state to induce MMPs production.

### 6.3. Role of N-Glycosylation in CD147 Maturation

*N*-linked glycosylation plays important roles in many aspects of intracellular protein biosynthesis, such as protein folding, quality control, oligomerization and transport. However, the molecular mechanisms remain unclear. Exploring the role of the conserved glycosylation sites leads to a better understanding of the underlying mechanisms. Importance of certain *N*-glycosylation sites in protein maturation and activity was found in Tyrosinase related protein (TRP) family and α5 subunit of integrin [69,116].

As a transmembrane protein, both CD147 on plasma membrane and a small fraction of extracellular secreted CD147 are capable of inducing MMPs. Current studies suggest two possible mechanisms through which CD147 are secreted from cell surface: vesicle shedding and proteolytic cleavage, which produce full-length soluble CD147 and CD147 lacking transmembrane or cytoplasmic domain, respectively [117–120]. As mentioned above, CD147 on the plasma membrane and in cell conditioned medium are fully glycosylated mature form [30,53], implying that the glycosylation of CD147 may be essential for its translocation to the cell surface. Site-specific mutagenesis experiment verifies that only initial *N*-glycans on Asn152 play a vital role in the quality control of CD147 in the ER and determine its cell surface expression and activity. We reason that *N*-glycans on Asn152 may directly participate in the protein folding or is significant for the interaction between CD147 and partner proteins in protein folding, such as calnexin, calreticulin, and BiP [53]. Considering the high conservative property of the three sites across species, we believe that all the glycosylated sites may be vital for CD147. The functional diversities of each site remain to be clarified in the future.

Aberrant glycosylated CD147 by mutating the glycosylated site Asn152 retained in the ER are degraded through ER associated protein degradation (ERAD) pathway [53]. However, under normal circumstances, LG-CD147 is also superabundant owing to its continuous transcription [121]. This noticeable overproduction of CD147 ensures the interaction of CD147 and other proteins and the exertion of protein functions. For example, the association between CD147 and MCTs facilitates MCTs assembly and trafficking to the cell surface, which are only up-regulated during cell adaptation to glycolysis [58,94,121]. Tyler *et al*. further elaborated the ERAD pathway of the excessive LG-CD147. By mass spectrometry analysis they identified endogenous LG-CD147 in the ER as a substrate of proteasome, which was degraded via OS-9/SEL1L/Hrd1 pathway, a possible fundamental degradation manner of CD147 [54].

### 6.4. Role of N-Glycosylation in the Interaction of CD147 and Other Proteins

Glycosylation is involved in protein interaction. For example, the *N*-glycosylation of CD44 is crucial for its binding to E-selectin, and the *O*-glycosylation of P-selectin glycoprotein ligand-1 (PSGL-1) enhances the binding of PSGL-1 to E-selectin and P-selectin [122,123]. It has been discussed previously that many molecules regulate the maturation of CD147. On the other hand, CD147 glycosylation also regulates its association with its partner molecules. Kato and co-authors reported that CD147 on the cell surface of neutrophils bound to E-selectin during leukocyte infiltration in the renal inflammation, and CD147 glycosylation is essential for the interaction since tunicamycin treatment to inhibit the *N*-glycans of CD147 from HL-60 cells reduced this interaction [27]. However, Tang’s study demonstrated that deglycosylation of CD147 resulted in increased interaction between CD147 and caveolin-1, suggesting that CD147 glycosylation interferes its interaction with caveolin-1 [32]. The possible role of the *N*-glycosylation of CD147 in its interaction with other proteins, such as integrins, MCTs, and cyclophilins, remains to be investigated.

As shown in the crystal structure of CD147 [42], the unique domain arrangement, which is responsible for the flexibility to interact with different ligands and diverse dimerization manners, is one structure basis for its multifunction character. At present, we conclude that another contributing factor is the distinct glycosylation feature of the molecule. Post-translational modification of CD147 modulates its biological functions in many aspects, including affecting protein maturation and translocation to the cell membrane, facilitating oligomerization and, hence, promoting MMPs production and tumor metastasis. In addition, *N*-glycans of CD147 also participate in the interaction with other proteins and exert corresponding biological effects.

## 7. Conclusions

As a highly glycosylated transmembrane adhesion molecule, CD147 plays a comprehensive role in many physiological and pathological processes. The applications of NMR, X-ray diffraction and structure-function studies by site-directed mutagenesis have illustrated the structure of CD147 and the mechanisms of the interaction of CD147 and other molecules, as well as CD147 itself, which underlies its various functions. Meanwhile, in this post-genomic era the studies on the characteristics of CD147 *N*-glycosylation highlight its importance. Given that the structure of the oligosaccharides and their functions have only been partly unveiled, further studies are required to elucidate molecular mechanisms underlying the effects of *N*-glycans on the functions of CD147 in cancer biology, to disclose the distinct oligosaccharides structures on its three glycosylation sites and their respective functions and to confirm whether aberrant glycans on CD147 could be used as a marker to predict clinical prognosis of cancer or drug resistant response of cancer therapy. We envision that this knowledge will provide direct and convincing evidence for the development of novel therapeutic perspectives, such as antibody drugs and small molecule antagonists targeting aberrant *N*-glycan structures in the treatment of CD147-associated diseases. It is noteworthy that Licartin, the 131 I-labeled CD147 mAb developed in our laboratory, has been applied safely and effectively in the treatment of patients with hepatocellular carcinoma [124,125]. It is reported that 41% of antibodies to a cancer cell recognized carbohydrate epitopes [126], thus, whether the sialyl Lewis structures and other carbohydrate components of CD147 glycosylation are involved in the interaction between Licartin and CD147 awaits investigation. More innovative drugs specifically targeting CD147 with higher efficacy will be discovered in the future.
